# Air pollution-related health impacts from domestic waste burning and associated interventions: the merits of a traditional versus machine learning scoping review methodology

**DOI:** 10.1007/s10661-026-15376-0

**Published:** 2026-05-18

**Authors:** Thandi Kapwata, Candice Webster, Wen Guan Gavin Chen, Muhammad Ibrahim Suder, Devon Jarvis, Alex Vogt, Nomfundo Mahlangeni, Tracey Laban, Natasha Naidoo, Pierpaolo Mudu, Kerolyn Shairsingh, Caradee Y. Wright

**Affiliations:** 1 Climate Change and Health Research Programme, Environment and Health Research Unit, South African Medical Research Council, Pretoria, South Africa; 2 Department of Environmental Health, University of Johannesburg, Johannesburg, South Africa; 3 School of Computer Science and Applied Mathematics, University of the Witwatersrand, Johannesburg, South Africa; 4 Machine Intelligence and Neural Discovery Unit, University of the Witwatersrand, Johannesburg, South Africa; 5 Department of Environment, Climate Change and Health, World Health Organization, Geneva, Switzerland; 6 Department of Geography, Geoinformatics and Meteorology, University of Pretoria, Pretoria, South Africa; 7 Environment and Health Research Unit, South African Medical Research Council, Cape Town, South Africa

**Keywords:** Air pollution, Artificial intelligence, Waste burning, Waste policies, Scoping reviews, Big data, Biostatistics, Computational science, Environmental health, Deep learning

## Abstract

Regularly updating repositories of air pollution impacts on health is essential for evidence-based policies, interventions, and progress monitoring. However, this is often time-consuming and labour-intensive. Automation can ensure up-to-date evidence; reduce retrieval, screening time, and costs; and make information accessible to non-academic stakeholders. This study investigated whether a machine learning approach can perform any stages of a traditional scoping review on the health impacts, policies, and interventions related to air pollution due to domestic waste burning. A traditional approach was conducted in parallel with machine learning methods enabling a comparison of the efficiency and quality of the partially automated approach against the manual review. Findings from the two approaches were compared and the final set of included articles were considered for (1) reported impacts of waste burning on health outcomes and (2) recommendations on solutions and interventions to prevent/reduce adverse effects on health from waste burning. We found a range of health impacts associated with waste burning, including low birth weight, hypertensive disorders of pregnancy, adverse respiratory outcomes like asthma and wheeze, cancer risk, and mortality. Few studies proposed solutions or evaluated the effectiveness of interventions. The ML approach showed a tendency towards false positives, which are preferable to false negatives (where relevant papers were excluded). Results showed that the model can conduct initial searches and decisions for the review. However, the articles included in the model should be screened manually for final acceptance. Therefore, we propose a hybrid approach be used until the automated model can be further refined.

**Supplementary information:**

The online version contains supplementary material available at 10.1007/s10661-026-15376-0.

## Introduction

Domestic waste burning is a common practice in many countries around the world (Karanasiou et al., 2021) and is associated with the emission of air pollutants (Ramadan et al., 2022). Open burning of waste produces harmful air pollutants which reduce air quality and negatively impact human health, in both short and long terms (Karanasiou et al., 2021; Lelieveld et al., 2015). Here, domestic waste burning is defined as open waste burning of dumped residential/domestic waste where the latter is defined as any type of garbage, trash, refuse or discarded material, either located in a backyard, or in the street or unofficial dumping sites in a community. The definition excludes waste in landfill sites, municipality-managed or owned dump sites, the burning of crop residues and the burning of street food. An example of domestic waste burning is the burning of garden plant waste that has been linked to the emission of polychlorinated dibenzo-p-dioxins (PCDDs) and polychlorinated dibenzofurans (PCDFs) (Hedman et al., 2005; Noblet et al., 2021).

Given the significant health risks posed by domestic waste burning, it is crucial to have evidence-based policies, interventions, and monitoring activities to inform prevention of waste burning. The number of publications on this topic has increased substantially, with 103 new publications on the topic in 2021 compared to 2018, as listed on PubMed. As the volume of research grows (Dhital & Rupakheti, 2019), there is a pressing need for an efficient approach to screen and synthesize information on domestic waste burning and its associated air pollution health challenges. This study was aimed at addressing this need by investigating whether a machine learning (ML) approach can mimic the performance of a traditional scoping review in identifying, selecting, and synthesizing relevant studies, ultimately supporting the development of timely and effective interventions and policies.

ML is an artificial intelligence technique that uses computational algorithms and statistical models to analyse complex relationships between variables in a dataset (Vokinger et al., 2021). ML can be divided into supervised, unsupervised and reinforcement learning. Supervised learning uses algorithms to find a mapping between data points and their corresponding labels to classify or predict future outputs (El Bouchefry & de Souza, 2020; Yalug et al., 2021). Unsupervised learning uses algorithms to analyse and cluster unlabelled datasets (essentially raw data before labelling) and finds patterns in the data. Reinforcement learning trains the algorithm to make decisions and predict the next action that gives maximum reward through a series of trial and error (El Bouchefry & de Souza, 2020; Yalug et al., 2021). ML has been used as a tool to mine policy documents (Biesbroek & Delaney, 2020; Firebanks-Quevedo et al., 2022) and to conduct automated scoping reviews (Khalil et al., 2022; Mozgai et al., 2023). It is applied to accelerate various stages of the scoping review process, including searching, screening, and data extraction.

O’Mara-Eves et al. highlighted that since an experienced reviewer can take 30 s to several minutes to screen a citation, the task of screening 10,000 citations becomes more substantial (O’Mara-Eves et al., 2015). In their review of 44 studies, they employed support vector machines, latent Dirichlet allocation, *k*-nearest neighbour, and active learning (O’Mara-Eves et al., 2015). Also, while the reviewer is likely to have the ability to evaluate the evidence based on their expertise and experience, this assessment is inherently subjective (Zheng et al., 2010). Conversely, algorithmic approaches are more consistent but pose a potential difficulty in understanding why a particular decision is made. However, the use of automated approaches is a relatively recent development, and more evidence is required to support the feasibility of using ML for scoping reviews. Here, we investigated whether a ML approach and a traditional scoping review were comparable tools to screen, identify, and extract information from articles that contained relevant findings of the relationship between domestic waste burning, air pollution and related health impacts. Our paper (1) presents recent evidence of air pollution-related health impacts from domestic waste burning and interventions and policies aimed at reducing the adverse impacts using a traditional scoping review and (2) evaluates whether a ML approach is a feasible tool to automate the process of knowledge synthesis to regularly update evidence with the aim of improving public health by informing practice, programmes, and policy related to health and environmental issues involving waste burning.

## Methods

**Fig. 1:**
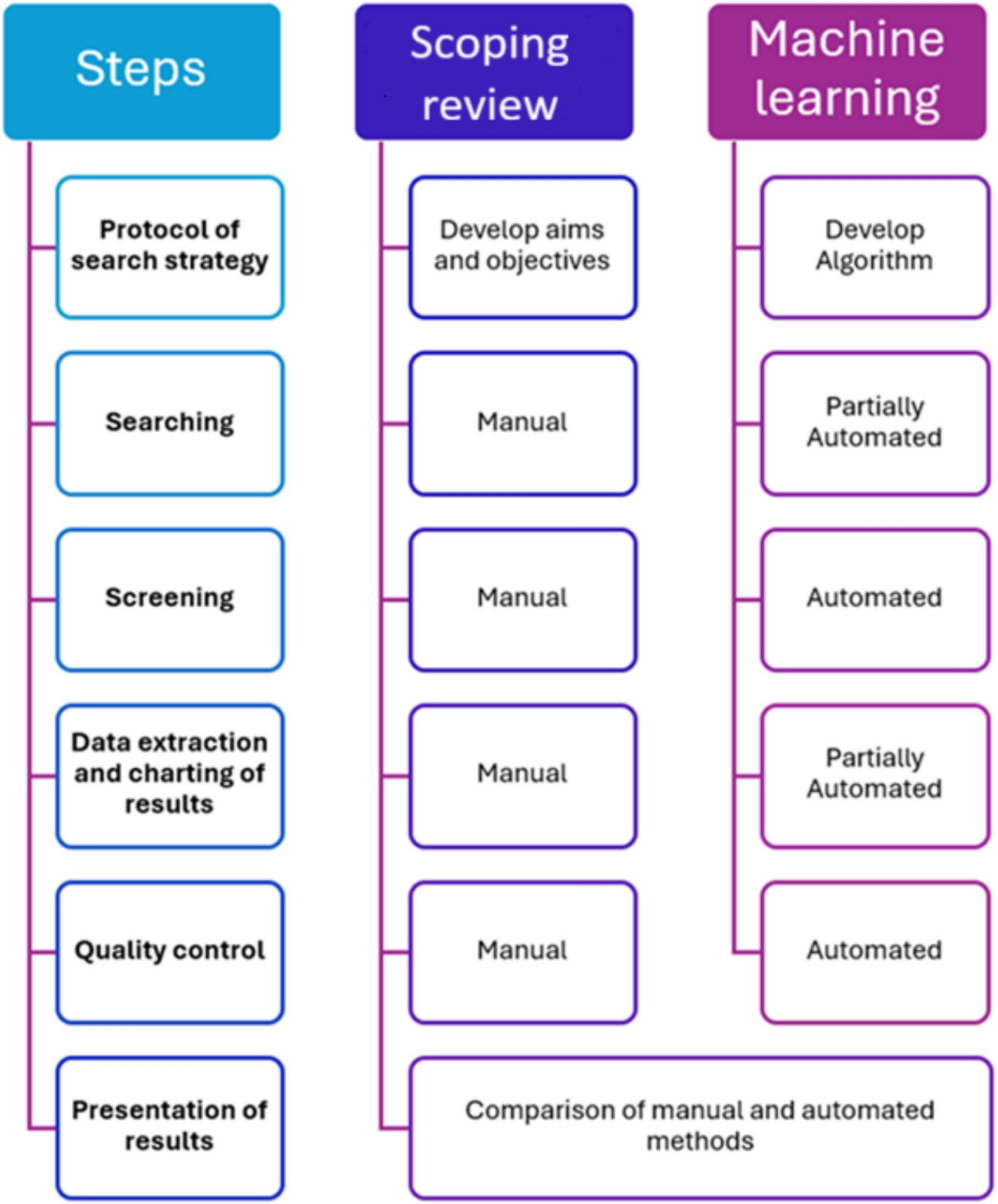
A diagram showing the comparison of the steps involved in the scoping review versus the ML algorithm

The traditional scoping review and the partially automated ML scoping review were conducted during the same period. Figure 1 shows the steps involved in each approach.

### Traditional scoping review

The Johanna Briggs Institute framework adapted from Arksey and O’Malley (2005) was used to conduct the scoping review (Arksey & O'malley, 2005). This framework included the following steps: (i) developing the objectives and questions; (ii) search strategy; (iii) inclusion criteria; (iv) extraction and charting of results; (v) consultation; (vi) presentation of the results; and (vii) discussion, conclusion, and implications for research and practice. This scoping review was reported following the Preferred Reporting Items for Scoping Reviews and Meta-Analysis (PRISMA).

### Partially automated machine learning approach (described in more detail below)

In parallel, we developed a partially automated ML algorithm to search, screen and classify publications and reports according to whether they comprise text relating to health impacts and interventions and policies around domestic waste burning and air pollution and related health impacts. Finally, we compared the document retrieval capacity and classification for the manual scoping review and our ML approaches.

### The scoping review procedure

#### Eligibility criteria

We followed the PECOS framework (Population, Exposure, Concept/Context, Outcome and Study) in identifying our eligibility criteria. Studies eligible for inclusion will have to meet the following criteria:

- Population: The targeted population that was non-specific, although special attention will be given to groups who are vulnerable to the impacts of waste burning and air pollution, e.g., infants and children, older persons, and people with pre-existing/chronic diseases.
- Exposure: Studies that assessed environmental factors, agents or exposures related to waste burning and air pollution, i.e., particulate matter, and black carbon, among others.
- Concept/Context: Literature on waste burning and/or air pollution in rural, urban, and informal settlements.
- Outcome: Outcomes related to waste burning and/or air pollution emissions, air pollution-related morbidities (including cardiovascular, cerebrovascular and respiratory diseases, neurological and cognitive disorders, bone conditions, and cancers), or mortalities; in addition, studies reporting on solutions for adaptation, mitigation, or interventions.
- Study designs: Primary studies (cohort, cross-sectional, longitudinal, ecological, case studies, and interventions) that investigated health outcomes associated with domestic waste burning and air pollution.

#### Exclusion criteria

Articles were excluded for the following reasons using the PECOS framework:

- Population: No populations excluded.
- Exposure: Studies that did not assess environmental-related domestic waste burning and/or air pollution such as studies on non-domestic activities, landfills, pesticides, and heavy metals, among others. We also excluded burning for street food preparation, cottage industries where people work from home and burning waste from commercial activities.
- Context: Commercial activities. We decided not to include incineration as we did a trial run and a search with this work included commercial incineration which is not a term that is used in the domestic/community setting.
- Outcomes: Assuming that reduction in air pollution leads to an improvement in health, any study not reporting on the impacts on exposures/emissions or human health or adaptations/interventions related to human health and air pollution/waste burning.
- Study designs: Studies investigating exposures not related to waste burning and/or air pollution. We did not include conference abstracts, books and book chapters (since these are not always peer-reviewed) and book reviews, protocols, and animal studies. We did not include studies in any language besides English.

### Search strategy

Working with an information specialist, we first conducted a pilot search strategy to enhance our search precision. A comprehensive literature search was conducted after the pilot search in five relevant electronic databases: Web of Science Core Collection (accessed via Web of Science); Scopus (accessed via Elsevier); CAB Abstracts (accessed via Web of Science/OVID); MEDLINE; and EMBASE. In addition, reference lists of eligible articles were manually searched for additional articles relevant to the scoping review. The databases searched for relevant articles using text words (Table S1) and Medical Subject Headings (MeSH) (Table S2) (unique to each database) as part of the search strategy for domestic waste burning, air pollution, and solutions to prevent adverse associated health outcomes, as defined above, concerning human health, from the inception of the database. The text words and MeSH terms included terms related to:

- Exposures: air pollution, air quality, actions on clean air quality, ambient air quality standards, emissions, indoor air quality, household air pollution, open dumping, waste, etc.
- Outcomes: health impacts, mortality, morbidity, diet, nutrition, mobility, injury, health risk, exposure, diseases, wellbeing, and mental health.
- Solutions: policy, interventions, implementation, climate adaptation, air pollution mitigation, air quality management, adaptive capacity, and resilience.

Results of the searches, i.e., records and deduplication, were managed in EndNote and Rayyan.

### Study selection

Three review authors (TK, CW, and CYW) independently screened the titles and abstracts of articles identified in the literature search for relevance against the inclusion and exclusion criteria listed in this protocol. Subsequently, the authors independently screened full texts of potentially eligible articles for inclusion and exclusion, as per the stated criteria above. The authors provided reasons for excluded studies. During the study selection phase, disagreements between two authors were resolved through discussion with a fourth author (NM) who arbitrated when a consensus was not reached.

### Data charting and synthesis

Three authors (TK, CW, and NM) independently extracted data using a piloted data extraction form in Microsoft Excel. Data extracted from eligible studies included the first author’s last name, year of publication, the country where the study was conducted, study design, study duration, participant characteristics, data source, type of air pollutants, and key findings (impact on human health and pathways), solutions in the form of interventions, recommendations and policies to reduce waste burning and improve health outcomes, and whether the article was open access or subscription only. 

Data synthesis involved analysing findings from the identified studies using high-level domains pertaining to human health outcomes. Themes were aggregated to synthesize overall findings for the manual scoping review and the ML automated review. We also considered the differences in the retrieved studies from the manual and ML approaches and, if there were differences, what biases may be identified to help inform lessons learnt.

### The machine learning algorithm

Large language models (LLMs), such as BERT (Alaparthi & Mishra, 2020; Cañete et al., 2023) and DistilBERT (Sanh et al., 2019), are Transformers trained on large amounts of text to learn general patterns in language. A key feature of these models is the self-attention mechanism (Chorowski et al., 2015; Liu et al., 2021; Vaswani et al., 2017), which allows the model to assign importance to different parts of the input text when making predictions. This enables the model to understand contextual relationships between words.

The various stages we employed formed a “curriculum” (Bengio et al., 2009) which exposed the model to various tasks in sequence of increasing difficulty. This had the benefit that earlier tasks in the curriculum were typically easier to collect data for, significantly increasing the efficiency of learning by targeting certain properties with each stage. The second core concept which we employed was model “pre-training” (Erhan et al., 2010). This fitted in naturally with the notion of a curriculum and expressed the fact that Transformer models exist which were trained to generate language and were shown a huge amount of unlabelled data to acquire this skill (OpenAI, 2023). Consequently, these models already had intrinsic representations of natural language, such as grammar, syntax, and semantics (Erhan et al., 2010). Fine-tuning was a subsequent step where the model was trained on a smaller, labelled dataset specific to the task of interest, allowing it to adapt to the requirements of that task (Sanh et al., 2019). Finally, when the model was fine-tuned on a task with a different structure to what it was originally used for, it was termed “transfer learning” (Raffel et al., 2020). By transferring knowledge from the pre-training stage, the model can be fine-tuned with relatively little task-specific data (Li et al., 2019; Rasmy et al., 2021a, 2021b). When the target domain (the source of the data) differs from the data used in pre-training, fine-tuning can help the model adjust to domain-specific terminology and structure (Ganin & Lempitsky, 2015). With these concepts in mind, our pipeline consisted of two fine-tuning steps: (1) a transfer learning step (first fine-tuning) and (2) a domain adaptation step (second fine-tuning). We note that this intermediate transfer-learning step is distinct from biomedical-domain pre-training in models such as BioBERT or PubMedBERT, which are trained on very large biomedical corpora. Our intention here was not to reproduce such pre-training with a small dataset, but to introduce an explicit auxiliary objective that contrasts epidemiological/clinical versus non-epidemiological/non-clinical writing prior to topic-specific domain adaptation. In practice, this serves as a curriculum stage that biases the representation towards biomedical-style discourse and terminology, with the aim of improving sample efficiency in the final fine-tuning stage where expert-labelled examples that match the scoping review inclusion criteria are limited. Additionally, a model automating the scoping review needs to be able to handle language from multiple domains, not just medical language, particularly where the review also considers policies and interventions.

Thus, we chose to begin with a general and small foundation model (DistilBERT) and train for domain-specific language rather than begin with a medical model and train it to ignore all alternative fields.

Specifically our pipeline was as follows:

- We began by loading a pre-trained DistilBERT model (Sanh et al., 2019). This model had been trained on large-scale English text corpora and provided general-purpose language representations. Importantly, this model was still small enough that fine-tuning with a single graphics processing unit was plausible (we comment on our compute usage, below).
- We then performed an intermediate fine-tuning step using a binary classification task to distinguish between epidemiological/clinical and non-epidemiological/clinical papers. This step also transfers the knowledge of producing natural language from the DistilBERT and trains it to classify papers (changing the task of the model).
- Next, we fine-tuned the model on a small, expert-labelled dataset of papers that met the scoping review inclusion criteria. This step calibrated the model to the specific topic of the review using domain adaptation from the general epidemiological/clinical classifier towards the exact topic of focus. The need for labelled data at this step was why a smaller scoping review was still necessary within the larger pipeline. The question then was how many labelled data points were required for our model to achieve good performance (how much workload could be removed from the researcher). In practice, this expert input constitutes the primary form of “guidance” given to the model.
- During inference (using the model to make predictions), we divided each paper into overlapping segments to fit within the model's input length. We applied the model to each segment and averaged it across all segments to produce a single aggregated prediction. The final label for the paper was determined by selecting the class with the highest average probability score. Intuitively, this was synonymous with our model “reading” portions of the text and gradually deciding on the class label.

**Fig. 2:**
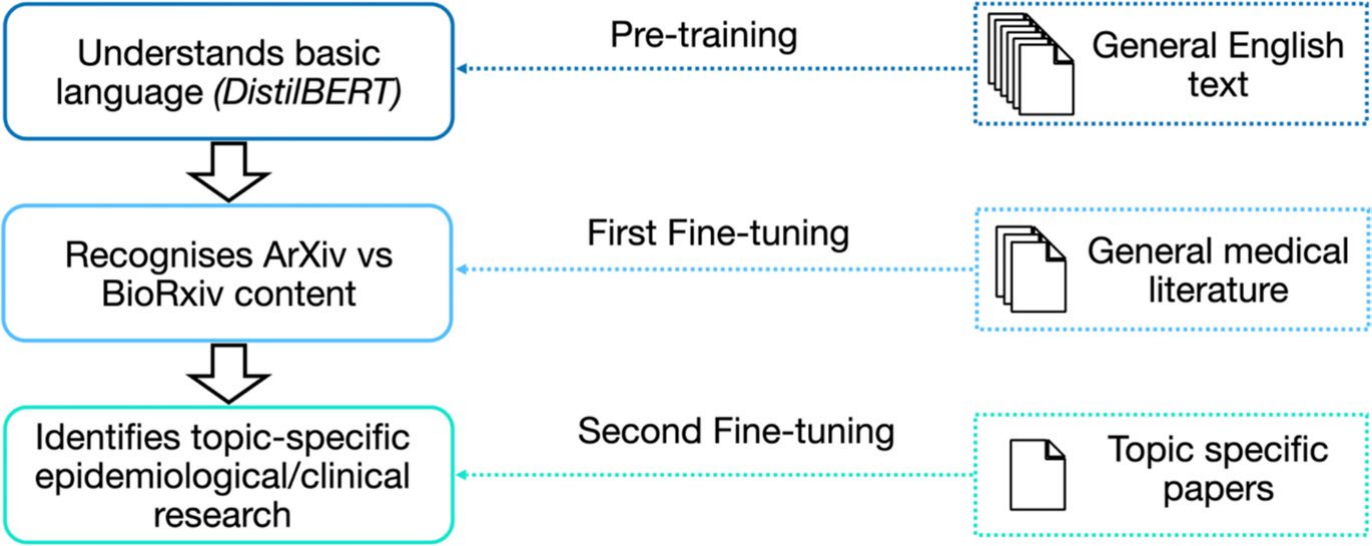
Three-stage training process: pre-training on general English text, transfer learning using general medical literature (first fine-tuning), and domain adaptation on topic-specific papers labelled by experts (second fine-tuning)

Importantly, the two individual fine-tuning steps form a curriculum that allows the model to first learn epidemiological/clinical language before being given a specific epidemiological/clinical domain of focus. This had the added benefit that finding data was much easier for an epidemiological/clinical versus non-epidemiological/clinical classifier than finding data that fit the scoping review inclusion criteria. In our case, we downloaded papers from ArXiv and BioRxiv and labelled the papers as medical and non-medical based purely on the basis of where they were downloaded. In the “Experiments” and “Results” sections, we performed an ablation on this initial fine-tuning step and found that there was a noticeable benefit from using the curriculum. The overall training pipeline is illustrated below in Fig. 2.

## Experiments

### Influence of training data sample size for the domain adaption step

To determine the capabilities of our pipeline to assist with scoping reviews, we conducted three experiments. The first aimed to determine how many exemplar papers the transformer needed to see before its performance saturated (i.e., there is no further benefit to showing the model more papers). The lower the saturation point, the less work would be required by a researcher when performing the scoping review. Thus, we trained our pipeline with a varying number of papers from the manual scoping review and evaluated its final performance. To increase the amount of labelled data available for training, the training set was constructed from papers labelled during the initial title and abstract screening stage of the manual scoping review, while the held-out test set was drawn solely from papers labelled during the final full-text screening stage. These sets were mutually exclusive. This allowed us to expand the training data while reserving a stricter full-text-labelled set for final evaluation. We trained our model on 4, 8, 10, 12 and 40 papers with a balanced split of “Yes” and “No” class labels. In this case, “Yes” meant that the paper met the inclusion criteria for the manual scoping review and “No” meant that the paper was excluded. Thus, in this balanced case only half of the papers used to train the model were actual examples of papers that met the criteria. We repeated the full training process multiple times, starting from different random seeds and reported the mean and standard deviation. From this experiment, we were also able to determine the likely performance on the model as the final accuracy was evaluated on a held-out, “test” set of papers which the model was not shown during training. This gave us a measurable indication of how well we can expect the model to perform in practice.

### The impact of successive fine-tuning steps

Our second experiment removed the medical versus non-medical fine-tuning step and jumped straight to the waste burning detection fine-tuning. The aim of this ablation experiment was to determine whether the curriculum afforded by the additional transfer learning fine-tuning was effective at inducing successful learning. Once again experiments were repeated multiple times, and the means and standard deviations were reported.

### Deployment of model

Our final experiment then deployed our best-trained model on BioRxiv (trained using our full pipeline and transfer and sequential fine-tuning) and let it label some new papers which were not a part of its dataset. We collected 20 new papers from the BioRxiv website for this experiment. Ten papers were collected by searching for the term “waste burning,” and the other ten were randomly selected from all other categories to serve as general background papers. We then applied our best model to predict whether each of these new papers discussed waste burning. This qualitative study aimed to simulate the ultimate deployment of the model as it would be used in a practical scoping review. Importantly, this experiment served a different purpose to the testing dataset in the first experiments, as we did not have the ground-truth labels for these new papers up front. Thus, this second test set which is scraped directly from BioRxiv without any filtering by a medical practitioner provides a realistic simulation of actually deploying our model. Ideally the performance of the model on the test dataset was an accurate reflection of its final performance when deployed. However, many factors can result in even a new test set providing unrealistic results, especially with limited data like we had in the waste burning setting. For example, the datasets received from the medical practitioners had all been papers which were selected for initial review (placing the negative samples close to the decision boundary). This new test set is potentially more difficult for the model as it is only taken from BioRxiv where exclusions are most difficult to determine but also where the greatest degree of medical language would be encountered. Thus, this experiment can also shed light on any looming issues which may still hinder our model’s performance.

## Results

### Descriptive findings from the traditional review process

**Fig. 3:**
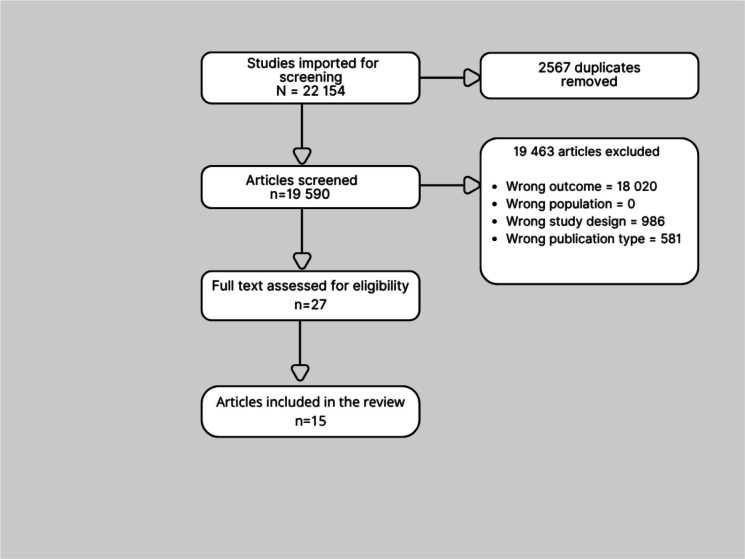
PRISMA diagram of screened and included articles from the traditional review process

Our initial search yielded a total of 22 154 articles. Among these studies, 27 met the study criteria. After full-text screening in the traditional manner, 15 studies met the eligibility criteria and were included in the review (Fig. 3).

### Descriptive findings from the ML review process

**Fig. 4:**
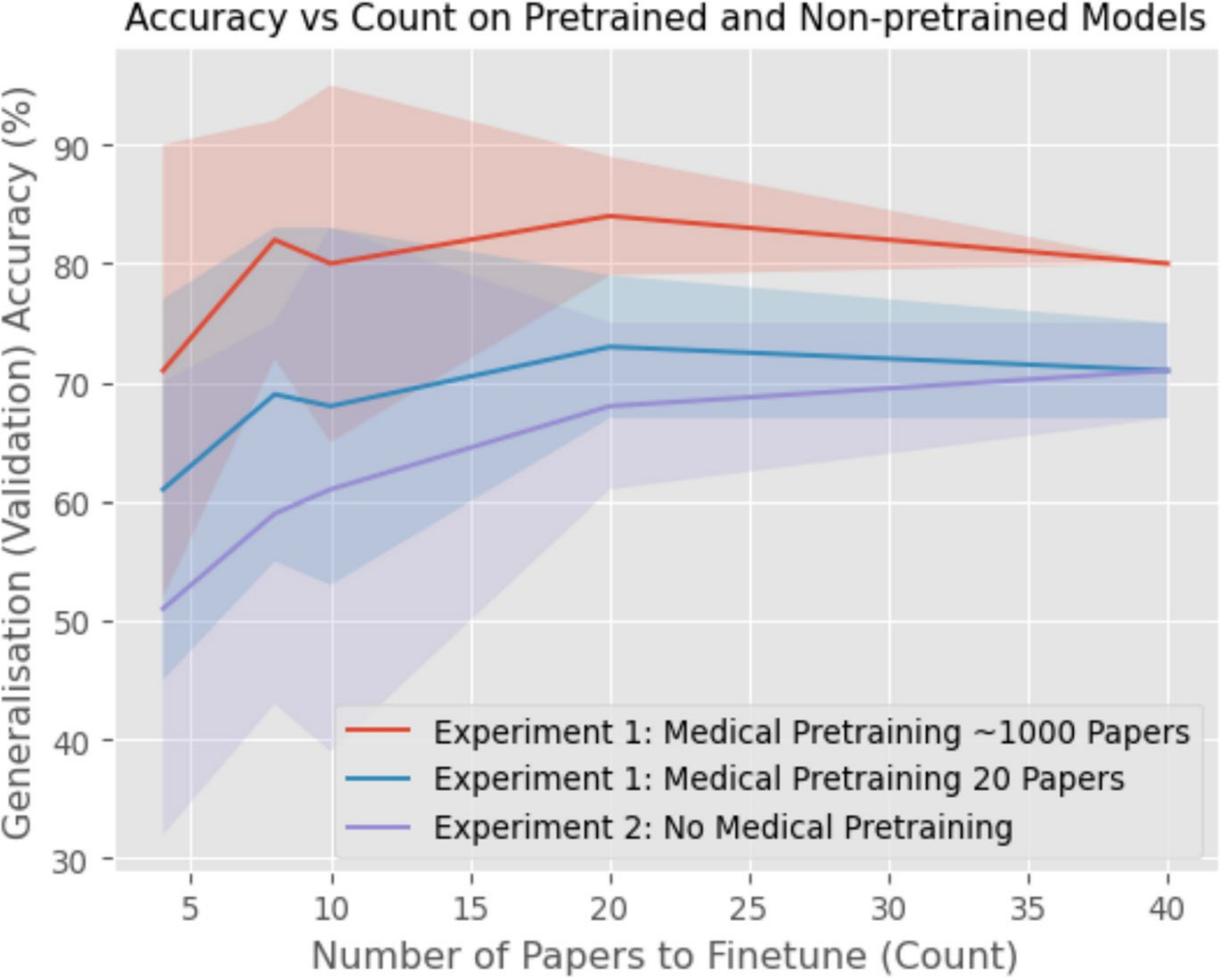
Accuracy of the full model (includes fine-tuning on general epidemiological/clinical domain classification) and ablation pipeline across various pre-training dataset sizes (where “medical pretraining” refers to the ArXiv vs BioRxiv pre-training). The full pretrained model significantly out-performs the ablation model, especially with fewer training samples. The full pipeline’s performance also saturates after approximately 10 papers, while the pipeline without general epidemiological/clinical fine-tuning requires significantly more data and keeps improving as a result. Results averaged over 11 runs and error bars depict two standard deviations

Figure 4 depicts the results of the full pipeline with varying numbers of papers used during training. The performance of the full pipeline began to saturate after approximately eight papers (only four examples of papers which met the inclusion criteria in the manual review). Furthermore, this accuracy was 84%. On a held-out labelled test set, this corresponded to a sensitivity of 67% and a specificity of 86%. This was a positive result as it demonstrated that the model was able to learn the task and perform well on a task which required many epidemiological/clinical terms to be parsed. Moreover, the number of papers required to reach this performance was very manageable for a researcher who only needs to find approximately five examples of papers which meet the selection criteria. Here, these exemplar papers are intended to represent a small expert-defined seed set and the model is then used to retrieve and rank additional papers that are similar to those known examples for subsequent verification, rather than requiring an initial corpus-wide manual screen to discover the first positives. Figure 4 also shows the effect of removing the initial fine-tuning (transfer learning step) where the model was first trained just to classify medical and non-medical papers. From this ablation we see that removing this pre-training led to a significantly lower accuracy and higher standard deviation in predictions compared to including pre-training. Importantly, we also did not observe that the performance of this pipeline saturated at a reasonable number of papers either, indicating that much of the task was just learning the epidemiological/clinical terminology and domain-specific language. This illustrates the benefit of introducing an explicit curriculum stage that contrasts epidemiological/clinical versus non-epidemiological/non-clinical writing, when training these models.

**Fig. 5:**
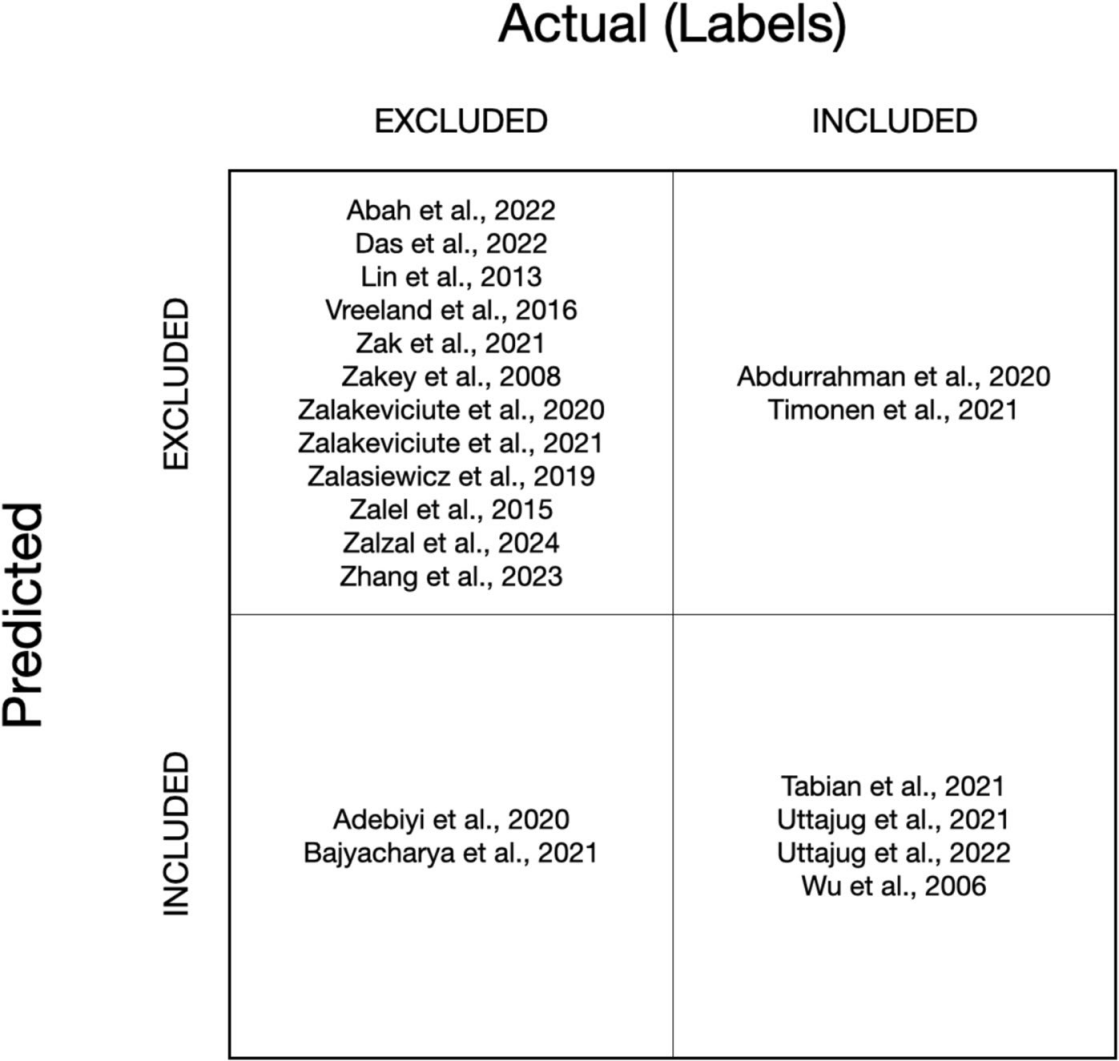
Paper-level confusion matrix for the held-out labelled test set. Each quadrant lists the corresponding studies, showing excluded papers correctly excluded and incorrectly included, as well as included papers correctly included and incorrectly excluded

To provide a transparent account of model errors, we additionally report a paper-level confusion matrix (Fig. 5) that lists the specific included and excluded papers that were correctly and incorrectly classified.

**Fig. 6:**
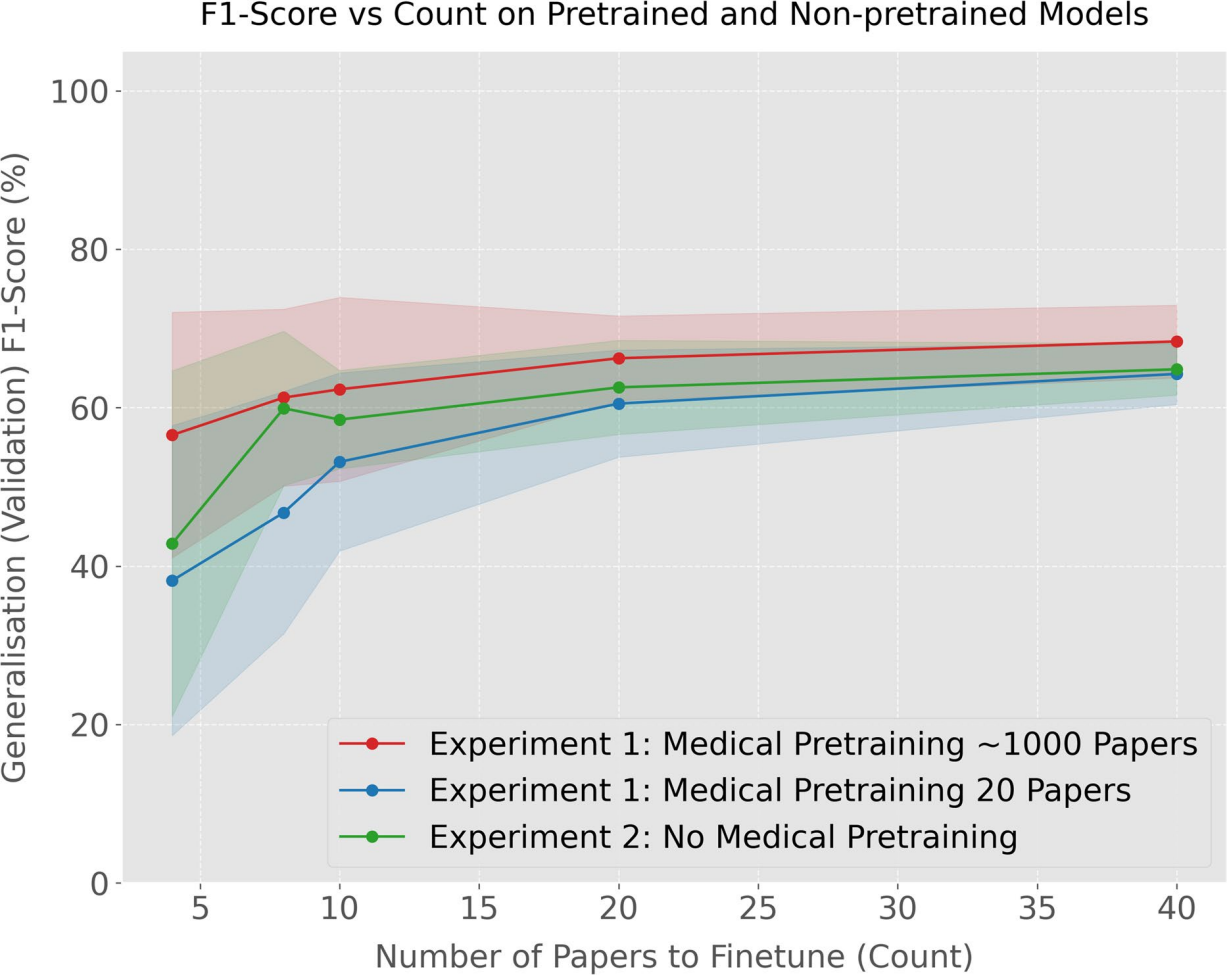
F1-score as a function of the number of papers used for fine-tuning for three model variants: the full model with medical pre-training (transfer learning step) on approximately 1000 papers, a reduced-pre-training variant using 20 papers, and a model without the intermediate medical pre-training stage. The full pre-trained model shows the strongest overall performance, while removing the intermediate pre-training leads to lower and less stable F1-scores, particularly at smaller second fine-tuning (domain adaptation step) set sizes. Shaded regions indicate variability across 5 runs

We also report the corresponding F1-scores across different numbers of papers used for fine-tuning (Fig. 6), which provide a useful complementary measure by jointly reflecting precision and recall rather than overall accuracy alone.

**Fig. 7:**
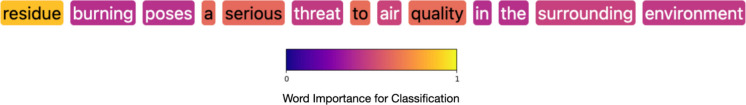
Example attention visualisation. The model highlights words related to waste burning as most important for classification. The colour scale reflects word importance. From this one example we can see that the model finds the word *residue* to be more informative for the inclusion criteria than other relevant words such as *burning* and *air quality*

We can also obtain some qualitative insight into the parts of the input that influence the model’s decisions by considering the attention maps (Chefer, Gur, & Wolf, 2021). These attention maps depict how much weight was being placed on each word in the input text by the model. Figure 7 depicts one such attention map on a chosen input text and shows that the model assigns greater importance to domain-relevant terms when making classification decisions. For example, in this instance, the model focused primarily on the word *residue*, even over other seemingly relevant words such as *burning* and *air quality*. This can allow a degree of transparency when determining the model’s decisions and, in cases when errors occur, lend insight into how to improve the model. In this case *burning* and *air quality* (as well as other filler words which we expect to be given low weighting) did not discriminate between inclusion and exclusion from the study to the same degree that *residue* did. Overall, this suggests that the model was learning meaningful representations aligned with the review criteria. However, we emphasise that this analysis should be interpreted by domain experts and does not constitute clear explanations from the model. It was not only an interpretability technique (Chefer et al., 2021), but also another benefit from using the Transformer model, compared to other machine learning approaches with less inherent interpretability (London, 2019). We therefore treat the attention visualisation as a qualitative interpretability aid for domain experts, rather than an objective or comparable score of word importance across segments or documents. The voting approach to making the full-paper decision also aids in this process as classifications where the votes are mixed can raise a flag that a human practitioner should intervene and use these attention visualisations to make a decision.

Finally, for the deployment experiment we found that our model correctly excluded all ten of the general background papers not taken from the “waste burning” search term. This was an important result as it demonstrated that the model was learning during the final fine-tuning stage on topic-specific data. If the first fine-tuning stage (transfer learning step) was dominating the model’s experience, then these papers would all be included in the model’s final decisions (since they come from BioRxiv—which receives the “Yes” label during initial fine-tuning). Thus, the curriculum afforded by the first general fine-tuning speeds up learning but the model was still able to learn to change the decision criteria during subsequent learning. For the papers from the “waste burning” search term, our model excluded three papers and included seven. This shows some discernment from the model on more difficult papers and an appreciation that not all waste burning papers were relevant to the scoping review. This was a sophisticated skill from the model and encouraging to see. However, based on the number of studies found in the manual scoping review, the model was likely including some studies which should be excluded. Subsequent verification determined that all 20 of the new papers should have been excluded and so the model tends toward false positives. However, in the context of a scoping review, false positives were far more manageable than false negatives were (where relevant papers were excluded) and the tendency for the model towards caution in this case was reassuring. Further, this behaviour is influenced by the epidemiological/clinical vs non-epidemiological/clinical pre-training which promotes the model to initially include all medical papers before being given a more specific task. However, it does demonstrate the need for researchers to verify the included papers from our model. However, this was likely to be far easier to do than conduct a full manual (traditional method) scoping review. We emphasise that this deployment exercise is intended to reflect practical use on newly retrieved literature where the prevalence of truly included papers is expected to be extremely low, and therefore, it primarily serves as a realistic stress test of false positives rather than a controlled estimate of overall performance. Improving specificity in such adversarial low-resource settings is an important direction for future work.

### Descriptive findings as well as socio-demographic and health outcomes

A total of 15 studies were included comprising 10 quantitative studies, four modelling-based assessments, and one qualitative investigation (Table 1). A full summary of the included studies is included in the supplementary material (Table S3). The studies spanned a diverse set of countries including Ghana, Kenya, Guatemala, India, Nepal, Poland, Indonesia, Costa Rica, and the Dominican Republic. This shows that most studies in our review were situated in low- and middle-income countries (LMICs) where open waste burning is a common practice due to limited waste services and associated infrastructure. Ghana was the most represented country accounting for 3 of the studies.

**Table 1:** Summary of included studies (*N* = 15)

	Number of studies	
Variable	*N*	%
Country/region of study		
Africa Costa Rica Dominican Republic Ghana Global Guatemala India Indonesia Indonesia/Philippines/Zambia Kenya Nepal Poland	1 1 1 3 1 2 1 1 1 1 1 1	
Type of study		
Quantitative Modelling Qualitative	10 4 1	
Health outcome		
Airway inflammation Asthma Birth weight Carcinogenic and non-carcinogenic risks Gestational diabetes Gestational hypertension Mortality None Preeclampsia/Eclampsia Respiratory infections Self-reported diarrhoea Urinary biomarkers Wheeze/trouble breathing	1 1 1 1 1 1 2 4 1 1 1 1 1	
Pollutant		
Black carbon Carbon monoxide Carbon dioxide Formaldehyde Methane Nitrogen dioxide Particulate matter 2.5 µg/m^3^ Particulate matter 10 µg/m^3^ Volatile organic compounds (unspecified)	3 2 3 1 1 1 7 3 1	

A comprehensive synthesis of the included studies identified four broad categories of adverse health outcomes associated with air pollution from domestic waste burning. These included respiratory-related impacts, adverse maternal and child health outcomes, and systemic and long-term disease risks. Air pollutants, especially particulate matter (PM), can penetrate deep into the lungs and enter the bloodstream where they adversely affect the respiratory system (Hamanaka & Mutlu, 2025; Ramos, 2022). Consequently, respiratory outcomes were the most investigated health effects. For example, a community-based study in Ghana found that domestic waste burning was associated with an increased risk of self-reported respiratory symptoms in both children and adults (*r* = 0.22, *p* < 0.05 and *r* = 0.25, *p* < 0.0001, respectively) (Boadi & Kuitunen, 2005). In Costa Rica, Werthmann et al. (2023) evaluated fractional exhaled nitric oxide (FeNO), a marker of airway inflammation, in women and children and observed that reported smoke exposure from waste burning was associated with higher odds of elevated FeNO in both groups (though estimates were not statistically significant), suggesting a plausible link between waste-burning smoke and airway inflammation in rural communities. Specifically, wheezing was strongly associated with increased FeNO (OR = 4.50, 95% CI: 2.25–8.99, *p* < 0.001), as was rhinitis (OR = 3.67, 95% CI: 1.81–7.35, *p* < 0.001) among women who participated in the study. In Nairobi, Kenya, Meme et al. (2023) found that children living in an informal settlement had greater exposure to multiple pollution sources and that self-reported exposure to “refuse burning near homes” was among the exposures significantly associated with increased risk of asthma-like symptoms (e.g., wheeze and trouble breathing), alongside other household sources such as mosquito coil burning and tobacco smoke.

The physiological changes which occur during pregnancy, such as increased oxygen consumption (~ 20%), elevated minute ventilation (40–50%), and increased cardiac output (~ 40%), increase the amount of pollutants inhaled and circulated, thereby increasing exposure (Aguilera et al., 2023). This is consistent with findings reported by two studies from Ghana. Amegah et al. (2012) found that garbage burning during pregnancy was significantly associated with low birth weight (LBW). The adjusted risk ratio was 2.95 (95% CI: 1.10–7.92, *p* < 0.05) for garbage burning alone and increased significantly to 4.16 (95% CI: 2.02–8.59, *p* < 0.05) when combined with charcoal use. In a follow-up study (Amegah et al., 2022), it was found that exposure to household air pollution (HAP) from biomass fuel doubled the odds of hypertensive disorders of pregnancy (AOR = 2.15, 95% CI: 1.05–4.43, *p* < 0.05), while garbage burning increased risk more than six-fold (AOR = 6.35, 95% CI: 2.43–16.58, *p* < 0.001). Gestational diabetes mellitus showed a positive association with HAP exposure, though not statistically significant (*p* > 0.05).

In Poland, household burning of municipal waste was found to produce high levels of particulate matter and toxic pollutants that exceed air-quality limits and are associated with respiratory, cardiovascular, cancer and systemic health risks for exposed populations (Kicińska et al., 2024). For example, burning polystyrene in household furnaces generated the highest cancer risk (CR) values of CR = 1.04E−01 for children and 2.60E−02 for adults (Kicińska et al., 2024). At a global scale, an estimated 270,000 annual premature deaths are attributable to PM₂.₅ exposure from open waste burning (95% CI: 213,000–328,000) (Kodros et al., 2016). At a regional level, 167,000 deaths annually across Africa were due to residential and trash-burning-related PM₂.₅, with 8% specifically linked to trash burning (Gordon et al., 2023).

Across the studies, social and behavioural drivers emerged as central determinants of domestic waste burning. Younger age groups (i.e., individuals between 18–29 years) were significantly more likely to burn waste compared to those ≥ 50 years (AOR = 3.65, 95% CI: 1.05–12.68, *p* = 0.042) (Choi et al., 2022). Similar findings were reported in Sudan where a decrease in age was found to be a contributing factor to burning household waste (Waleed Makki et al., 2023). Individuals with lower levels of education (≤ 8 years) were more likely to engage in open waste burning compared to those with more education (> 13 years, AOR = 2.63, 95% CI: 1.39–5.32, *p* = 0.007). Previous studies have shown that education has a significant impact on the choice of waste disposal options as those with higher levels of education have more knowledge of the detrimental effects of waste burning on the environment and on health (Adzawla et al., 2019; Kumara & Pallegedara, 2020). Ethnographic insights highlight that in low-income settings, inadequate waste collection services, unaffordable costs, and social norms are associated with the persistence of waste burning (Pathak et al., 2023; Turner et al., 2021). This is an important finding as it highlights that this practice is often a result of necessity as no other options are available to address waste.

## Pollutants measured in waste burning studies

The studies measured pollutants including particulate matter (either PM_2.5_ or PM_10_) and several gases, including volatile organic compounds. Data on these air quality indicators were collected using sensors, such as Purple Air (Meme et al., 2023) and MicroAeth Black Carbon aerosol monitors (Kearns et al., 2024). PM_2.5_ was the most measured pollutant (seven studies) because it is associated with the greatest proportion of adverse health effects related to air pollution globally (Atuyambe et al., 2024; Li et al., 2022; Southerland et al., 2022).

## Solutions to reduce waste burning

Among the reviewed studies, only a few implemented or evaluated interventions to reduce waste burning. A community-based intervention in Uganda mobilized neighbourhoods to reduce visible trash fires through collective action (Werthmann et al., 2023). This yielded a 24% reduction in burning that was sustained even months after the campaign ended—demonstrating a successful example of empirically validated community-driven behaviour change. In rural India, the use of experimentally tested improved waste burning devices (such as mesh baskets and water-heater stoves) was investigated. While not deployed at scale, these devices significantly lowered emissions of harmful pollutants such as benzene and 1,3-butadiene compared with open burning, suggesting harm reduction potential in transitional settings. Another study from Indonesia proposed the practice of integrated waste management, estimating a potential 42.8% reduction in emissions (Zaman et al., 2024). Several studies emphasized the need to expand waste collection infrastructure, while others advocated for public education and awareness initiatives and policy reforms—such as legislations targeting burning and regulation of toxic plastics to discourage open waste burning (Boadi & Kuitunen, 2005; Choi et al., 2022; Cruz et al., 2023; Pathak et al., 2023). These studies illustrate that effective interventions should be multi-faceted bringing together components such as community mobilization, technological substitution, and regulatory enforcement.

## Discussion

Most high-income countries have reduced the contribution of domestic waste burning to air pollution through effective regulation, reliable waste collection and disposal services, and well-established waste management infrastructure (Ryan et al., 2021). Therefore, the studies that met our inclusion criteria were mainly from LMIC regions including Africa, Asia, the Caribbean, Central America, and Eastern Europe. In many of the countries included in our review, open burning is the dominant form of waste disposal due to inadequate waste management systems, policies and practices; the lack of public awareness and effective public participation; and weak legislation and enforcement (UNEP, 2018). Our results show that there is a significant gap in evidence regarding the health impacts of air pollution caused by domestic waste burning, which is a concern given the range of adverse health outcomes identified. These included adverse pregnancy outcomes, respiratory conditions, increased risk of cancer and mortality. We also found very few studies that discussed interventions, which indicates a lack of actions geared towards reducing the environmental and health impacts of domestic waste burning. In addition, we identified a lack of awareness of the connection between waste burning and air pollution, with a pertinent disconnect between science, policy and practice.

Policymakers require regular evidence from local, interdisciplinary studies to quantify the health impacts of domestic waste and the associated impacts on reducing air quality to inform policies that move beyond simply prohibiting the practice but also target solutions that address the root causes. Furthermore, strengthening existing interventions that address this practice also requires information on the effectiveness, feasibility and acceptance thereof. However comprehensive evidence synthesis techniques such as scoping reviews are time and labour-intensive which can limit access to evidence in LMICs where this knowledge translation is crucial. An emerging body of evidence suggests that ML methods could make the review process more efficient (Roth & Wermer-Colan, 2023). Therefore, parallel to the scoping review, we conducted a partially automated process to compare the two approaches and assess its ability to make scoping reviews more accessible in resource-constraint environments.

To the best of our knowledge, this scoping review was the first to gather information to map and synthesize evidence on the use of ML algorithms to identify solutions to waste burning and air pollution-associated adverse health outcomes. The framing of this review emphasizes the need for integrated solutions that maximise co-benefits for health and well-being.

This work shows the potential of utilising recent advances in machine learning to assist with the labour-intensive nature of conducting a scoping review. Most practically, we demonstrated the importance of using a pre-trained model with a clear curriculum to obtain the best results—especially on resource constrained topics with limited published findings (such as the health impacts of waste-burning). In this case, epidemiological/clinal-domain knowledge was pretrained in an early stage of the pipeline and proved to be useful for training a reliable and accurate model on waste-burning detection. Qualitatively, we show an example attention map which demonstrates our model assigned high importance to words directly related to the topic during the classification process and even focuses on relevant discriminatory words more than highly relevant but common words. While this was a positive result, it also shows the interpretability of the model, which we believe will be of great benefit to practitioners in the future, aiming to understand the reason for their models’ decisions.

Given that during the deployment experiment, the model excluded 3/10 articles that were excluded from the final scoping review, we recommend that a mini-scoping review be conducted initially to train the model (approximately ten papers would be needed for the inclusion and exclusion labels each). During this training stage, the model can conduct the vast number of initial searches and decisions for the review. Finally, the articles included in the model should be screened manually for final acceptance as part of the partially automated scoping review. The attention maps from the model would likely be highly beneficial when interpreting the reasons the model chose to include the papers.

A primary weakness of ML models is their need for data and sensitivity to biases in the data (Bender & Friedman, 2018; Bender et al., 2021; Brown et al., 2024; Walter, 2024). Many prior works have commented on the resource requirements and extensive energy costs of training an LLM, which should be factored into any decision to deploy ML in practice (Li et al., 2024; Strubell et al., 2019). In this work, we used a single Nvidia GeForce RTX 3070 and while this is certainly not a negligible computational requirement, it is reasonable. For example, this is the typically required computing capability to play modern video games. Thus, this work shows that moderate computational requirements are sufficient for a reasonable scale model to partially automate scoping reviews. Moreover, we demonstrated the time-saving capabilities of automated scoping reviews, as our final model was trained in approximately 30–60 min on the GPU after the epidemiological versus non-epidemiological pre-training for approximately half a day. Thus, our entire pipeline can be trained on reasonable compute in about a day (ignoring the cost of pre-training the DistilBERT which is divided among all users, which is the point of training large foundation models). Overall, our study shows that automation techniques could significantly reduce the time required for evidence synthesis to support informed decision-making. 

Therefore, we show that a partially automated scoping review can improve the link between scientific evidence and policy actions by collating relevant articles in the current climate of rapidly increasing volumes of research outputs, especially in resource-constrained countries. It would also support regular updating of evidence to inform decision-making for the development or refinement of targeted interventions that reduce or prevent the adverse health impacts of domestic waste burning.

### Future research recommendations

Future research could explore the development of more sophisticated unsupervised ML algorithms that integrate emerging technologies, and longitudinal health and exposure studies to track the long-term impact of interventions based on ML findings. Our findings have global relevance, especially for LMICs, where the burden of air pollution is often highest. We also highlight the possible scalability of ML approaches in diverse geographic and socioeconomic contexts, ensuring that the solutions we identify are inclusive and applicable worldwide.

## Conclusions

The adverse health impacts of waste burning are evident from the studies included in this review. However, more studies from countries that commonly practise waste burning are needed to highlight the associated environmental and health risks. This would strengthen evidence-informed decision-making around sustainable waste management in LMICs. It is widely accepted that policies informed by research evidence are more effective. Therefore, we also showed that there are merits of partially automating the scoping review process to update sources from which to collate recent evidence of domestic waste burning, which is a significant public health risk in countries that already have high disease burdens.

## Supplementary information

Below is the link to the electronic supplementary material.

ESM 1 (DOCX 29.4 KB)

## Data Availability

Data is provided within the manuscript or supplementary information files.
