# Supplementary material for: Air pollution-related health impacts from domestic waste burning and associated interventions: the merits of a traditional versus machine learning scoping review methodology
Source: Environ Monit Assess. 2026 May 18;198(6):602. doi: 10.1007/s10661-026-15376-0 (PMC13183761; doi:10.1007/s10661-026-15376-0)
Supplement: Supplementary file 1 — (DOCX 29.4 KB) [file 10661_2026_15376_MOESM1_ESM.docx]

**Supplementary Material**

Table S1. Search terms for traditional search

| **Health outcomes** | AND | **Source** | AND | **Context** | AND | **Outcomes** |
| --- | --- | --- | --- | --- | --- | --- |
| Health OR wellbeing OR human health OR health impacts OR health risk OR mortality OR morbidity OR health hazard OR respiratory health OR public health OR disease |  | Waste burning OR biomass burning OR combustion OR open burning OR trash burning OR domestic waste OR urban waste OR open air burning OR uncontrolled burning OR barrel burning OR garbage burning OR refuse burning |  | Domestic OR household OR private OR community OR municipal OR cities OR residential OR backyard OR rural OR individual |  | Air pollution OR fly ash OR particulate matter OR dust OR aerosol OR pollution OR air quality OR emission OR trace gases OR atmospheric impact OR atmospheric pollutants OR black carbon OR smoke |

Table S2: ML-MESH terms

Search terms

| **Health outcomes** | AND | **Source** | AND | **Context** | AND | **Outcomes** |
| --- | --- | --- | --- | --- | --- | --- |
| Health OR wellbeing OR human health OR health impacts OR health risk OR mortality OR morbidity OR health hazard OR respiratory health OR public health |  | Waste burning OR biomass burning OR combustion OR open burning OR trash burning OR domestic waste OR urban waste OR open air burning OR uncontrolled burning OR barrel burning OR garbage burning OR refuse burning |  | Domestic OR household OR private OR community OR municipal OR cities OR residential OR backyard OR rural |  | Air pollution OR fly ash OR particulate matter OR dust OR aerosol OR pollution OR air quality OR emission OR trace gases OR atmospheric impact OR atmospheric pollutants OR black carbon |

**INPUTTED AS**:

( ( ( ( health OR wellbeing OR human AND health OR health AND impacts OR health AND risk OR mortality OR morbidity OR health AND hazard OR respiratory AND health OR public AND health OR disease ) ) AND ( waste AND burning OR biomass AND burning OR combustion OR open AND burning OR trash AND burning OR domestic AND waste OR urban AND waste OR open AND air AND burning OR uncontrolled AND burning OR barrel AND burning OR garbage AND burning OR refuse AND burning ) ) AND ( domestic OR household OR private OR community OR municipal OR cities OR residential OR backyard OR rural OR individual ) ) AND ( air AND pollution OR fly AND ash OR particulate AND matter OR dust OR aerosol OR pollution OR air AND quality OR emission OR trace AND gases OR atmospheric AND impact OR atmospheric AND pollutants OR black AND carbon OR smoke )

Table S3: Characteristics of included studies retrieved from the traditional systematic review process.

| **Author, year** | **Type of study** | **Country / region** | **Study year and duration** | **Participant characteristics** | **Type of pollutant and source** | **Health Outcomes** | **Key findings** | **Solutions** | **Access** |
| --- | --- | --- | --- | --- | --- | --- | --- | --- | --- |
| Amegah, 2012 | Cross-sectional study | Ghana | 2010 | Nursing mothers and neonates | None assessed; garbage | Birth weight | Garbage burning at home resulted in a 195% increase in the risk of low birth weight (LBW). High exposure from garbage burning was also associated with a 359% increase in the risk of LBW. | None | Open |
| Amegah, 2021 | Cross-sectional | Ghana | 2020, 3 months - June to August | Nursing mothers residing in Cape Coast and accessing postnatal services at the two selected health facilities. | Household Air Pollution (HAP) exposure information collected using a structured questionnaire. Derived an index of HAP exposure as follows: (1) mothers using liquefied petroleum gas (LPG) and / or electricity for cooking were classified as having no HAP exposure and served as reference category; (2) for mothers using biomass fuels for cooking. | Gestational diabetes, eclampsia, preeclampsia and gestational hypertension | About 8% of the participants reported burning garbage at home during pregnancy. HAP exposure due to garbage burning at home was associated with about six fold (AOR = 6.35; 95% CI: 2.43, 16.58) increased odds of HDP. Mothers exposed to both cooking and garbage burning HAP had about 13 fold (AOR = 12.89; 95%: 2.69, 61.67) increased odds of HDP compared to the referent mothers. | Increased vitamin D intake during pregnancy may ameliorate HAP exposure effect on maternal  disorders of pregnancy.  During antenatal care visits, mothers should be educated on the importance of minimizing HAP exposure during the period of pregnancy and also encouraging them to increase intake of vitamin D-rich foods. | Subscription |
| Bardales, 2023 | Cross-sectional study | Guatemala | 10-weeks (June to August 2019) | Household members 18 years and older | PM_2.5_, BC, OC, CO, and CO_2_; burned plastic waste | None | Among the people whose primary mode of disposal is burning, the quantified average amount of plastic waste that would have been burned per person per day was 2.66 × 10−2 ± 1.32 × 10−2 kg | Providing access to proper waste disposal programs in low-resource countries, especially in rural areas, could mitigate the amount of waste burned domestically and thus reduce indoor and outdoor air pollution from this source. | Open |
| Boadi, 2005 | Cross-sectional | Ghana | Not reported | Female heads of households over 20 | Not reported | Self-reported diarrhoea | Burning solid waste Is correlated with the incidence of respiratory health symptoms among both adults (i- = .25. p < .0001) and children (r ^ .22, p < .05). In the multivariate test of variance, solid waste burning showed a significant association with the incidence of respiratory infections in adults (p = .004, 95 % CI) and children (p = .01, 95 % CI). | An efficient solid waste management system remains an appropriate tool for achieving sound environmental health in Accra. | Open |
| Chaudhary, 2022 | Modelled | India | Not reported | None | 76 VOCs, CH_4_, CO, and CO_2_; burned trash | None | Legalizing waste burning and encouraging households to substitute traditional water heating fuels with dry plastic waste can reduce emissions from open waste burning and improve air quality. The highest air quality benefits can be achieved by implementing a scenario where rural biodegradable waste is managed by decentralized compost or by putting it into biogas plants. Non-recycled and non-collected mixed plastic and multi-layered packaging waste is used to replace traditional water heating fuels. | “Smarter” waste burning can result in net air quality improvements. Education and outreach material spreading the knowledge that burning dry packaging waste in a stove or water heater is less harmful to human health than using cow dung or wood in the same device can spread this practice. | Subscription |
| Choi, 2021 | Cross-sectional survey | Nepal | 3-12 February 2018 - survey; 15-27 February 2018 - PM_10_ | Household members 18 years and older | Particulate matter | Perceived health risks | 60% of people said they use personal protective items such as masks showing that behaviours for coping with air pollution were more active compared with perceived air quality. Although 40.6% of respondents treated domestic waste by open burning, a high level of education was a significant determinant of less open waste burning and a high level of perceived health risks due to air quality. | Greater attention and assistance are needed to inform people in developing countries of the contribution of open waste burning to air pollution and its associated health risks to change the practice. Additionally, it should be accompanied by an emphasis on the importance of adequate and appropriate services for the collection and disposal of waste to reduce air pollution and its health impacts. | Subscription |
| Gordon, 2023 | Modelling | Africa | 2017 | Not described | PM_2.5_ | Mortality | Transported air pollution from trash burning had only minor impacts on ambient PM_2.5_ concentrations outside of the region where the emissions originated from due to the small magnitude of this source. They found that 11,200 premature mortalities were attributable to ambient PM_2.5_ from trash combustion across Africa. Of the five regions, the largest number of PM_2.5_-attributable premature mortalities occurred in North Africa (∼5,000 deaths). | Trash and residential solid-fuel emissions may be mitigated more easily through financial investments in municipal trash collection programs and/or assistance programmes that help finance conversions from residential solid-fuel use to electric stoves (as well as electrification of rural/remote African regions in general). | Open |
| Kearns, 2024 | Cross-sectional | Guatemala | Between 2017 and 2022 | Adolescent girls | Personal exposure to PM_2.5_ using Triplex Personal Sampling Cyclone (Mesa Labs) and Casella Tuff Pro pump (Casella, Buffalo, NY, USA), black carbon (BC) - Black Carbon - Model AE51 microAeth Black Carbon aerosol monitor | Urinary biomarkers | Burning trash was reported to be the preferred method of household garbage management by 76% of participants and the relationship between burning plastic trash in an open fire was associated with elevated BPA concentrations with nominal statistical significance (62% difference; p = 0.07). Concentrations of PM_2.5_ were 2.31 times higher (p = 0.02) in participants who had burned trash in a burn pile, and 3.32 times higher (p = 0.007) in participants who said the burn pile contained plastic trash compared to participants who had reported not receiving the same exposures. Detectable levels of phthalate and BPA metabolites were measured in urine samples, some of which were higher than the reference population, and some of which were related to burning trash. | None | Open |
| Kicinski, 2024 | Cross-sectional | Poland | October to the end of April (7 months). | Not described | PM_2.5_, PM_10_, HCHO, TVOC and the type of fuel combusted | Carcinogenic and non-carcinogenic risk assessment | Combustion of municipal waste in household furnaces is a substantial source of organic air pollutants (TVOC and HCHO) and thus constitutes a dangerous exposure source to people living in nearby areas. It is vital to stop burning such materials as polystyrene, rubber and upholstery foam in household furnaces due to the drastically high health risk associated with this practice. | The solution to the existing situation is to develop a model for managing renewable energy resources in such a way that individual energy sources are used most efficiently, with the least social cost and in accordance with the assumptions of sustainable development and environmental policies. | Open |
| Kodros, 2016 | Modelling | Global | One year: 2010 | Modelled/estimated population data | Estimated PM_2.5_ concentrations from models | Global and country-level mortality rates due to waste combustion | An estimated 270 000 (5th–95th: 213 000–328 000) annual adult mortalities from waste combustion. Waste-combustion emissions are approximately 10% of the total ambient PM_2.5_ mortalities and 9% of the total ambient PM2.5 mortalities estimated in the GBD 2010.  High mortality densities were found (>300 deaths per 104 km^2^ yr−1) in cities such as Mexico City, Moscow, Johannesburg, and Rio de Janeiro. | None | Open |
| Meme, 2023 | Cross-sectional observational study | Kenya | January 2020 and November 2021, 23 months | Children aged ≤18 years attending schools, 2373 children recruited 1 277 in Mukuru (median age, IQR 11, 9–13 years, 53% girls), and 1 096 in Buruburu (10, 8–12 years, 52% girls) | PM_2.5_ concentrations were measured using Purple Air PA-II- SD sensors | Questionnaires on respiratory symptoms and environmental exposures i.e. symptoms of wheeze and trouble breathing (all in the last 12 months) | Wheezing was adversely associated with refuse burning within sight of the home. Refuse burnt within sight of home (n, %) 330 (30.2%) for Buruburu and 485 (38.1% for Mukuru with p <0.001. | None | Open |
| Pathak, 2023 | Qualitative study - ethnography | India, Indonesia, the Philippines, and Zambia | December 2021–July 2022 | In each community, ten ethnographic interviews were conducted with those regularly engaged in burning, waste activists, local authorities, and private landfill operators. | Community and household practices related to the open burning of plastics. | N/A | In Indonesia, open burning was seen as an option for those unwilling to pay the monthly collection fee Households burned their mixed wastes every two days.  Waste burning was also conducted at a community level by mutual agreements between households within a neighbourhood.  In Zambia, garbage collection services were also deemed unaffordable and collection was not frequent enough | 1. Many anti-litter campaigns emphasize the removal of litter as normative behaviour but do not provide effective, convenient means for disposing of this litter. Addressing the harms of the open burning of plastic wastes will require interventions at the level of not just disposal—the creation of affordable, reliable, and effective trash collection and end stage processing alternatives that build upon local capacities—but also at the level of production, through a reduction in the manufacturing and consumption of plastics. A turn toward greater reuse, the use of less toxic plastic additives, and the phasing out of forms of packaging (e.g., multilayer packaging and multi-polymer packaging) and plastics (e.g., polycarbonate) that cannot be easily recycled is also needed. 2. Reductions in the use of Styrofoam (polystyrene) or polyvinyl chloride (PVC) wherever possible, incentivized through higher taxation and caps on production 3. Local campaigns raising awareness about the toxicity of the ash left behind by open burning to prevent use of this ash as fertilizer 4. Extended producer responsibility schemes that task manufacturers with collecting and safely—and under regular monitoring—recycling or disposing of the post-consumer waste. | Open |
| Turner, 2021 | Cross-sectional | Dominican Republic | August and October 2014 | Parents and caregivers (18 years of age or older) of children receiving health services from a paediatric clinic | Waste burning (qualitative study, no pollutants measured) | Respiratory infections and asthma | Trash burning was identified as the main contributor to poor air quality.  Participants described trash burning as a common practice often necessitated by limitations of the municipal Trash collection system.  Many participants voiced unease about what kind of trash material was burned (including plastic and tire rubber) and the health effects that may have on people in the community. | Community-based initiatives in other regions of the Dominican Republic have shown promise in efforts to clean up informal dumping sites in neighbourhoods and nearby bodies of water, to educate students about inorganic / organic waste separation by implementing composting in schools, and to support more formal recycling programmes. | Open |
| Werthmann, 2023 | Cross-sectional | Costa Rica | Visit in 2019, 8 years post-partum | Pregnant women living less than 5 km from a banana plantation in Matina County, Costa Rica. | Evaluated smoke exposure based on self-reported frequency of waste burning in the community (Never, Some / Monthly, Weekly / Daily). | Fractional exhaled nitric oxide (FeNO) is a marker of airway inflammation. | Exposure to smoke from waste burning was associated with elevated FeNO in women and children, although these results were not statistically significant. Waste burning was associated with elevated FeNO markers. | None | Open |
| Zaman, 2024 | Modelled | Indonesia | 2021 | Residents who were burning  waste | CO_2_, CH_4_, and N_2_O,black carbon, estimated using IPCC 2006 Chapter 5: Incineration and waste open burning guidelines | None described | Centralized open waste burning was designed as an effort by the community to reduce open waste burning activities. Simulations showed that waste management planning reduced emission levels due to open waste-burning activities by approximately 42%. | The regulation on the ban on burning waste in households was launched by a government in 2008 and states that every person is prohibited from burning waste that does not meet the technical requirements of waste management. However, in practice, the enforcement of this prohibition has yet to be thoroughly conducted, given the lack of adequate infrastructure to manage waste, such as an efficient waste collection and processing system.  As a result, some people may feel that they have no other option but to burn their waste. The community still needs to be better educated regarding the importance of enforcing this ban. Therefore, there is a need for increased law enforcement, campaigns that educate the public on the negative impacts of burning waste, and improvements to waste management infrastructure through more suitable alternatives. | Open |
